# Network characteristics of emotional resilience, anxiety, and depression among Chinese adolescents and their gender differences

**DOI:** 10.3389/fpsyt.2025.1651506

**Published:** 2025-09-16

**Authors:** Chang Liu, Xiao-Xia Pi, Bo Liu, Li Zhang, Meng-Ying Luo, Yu-Jun Zhang, Xin-Feng Zhang, Suo-Cheng Nie

**Affiliations:** ^1^ Mental Health Center of Yangtze University, Jingzhou, Hubei, China; ^2^ Mental Health Institute of Yangtze University, Jingzhou, Hubei, China; ^3^ Department of Psychiatry, Jingzhou Rongjun Special Care Hospital, Jingzhou, Hubei, China; ^4^ Department of Psychiatry, Jingzhou Mental Health Center, Jingzhou, Hubei, China

**Keywords:** emotional resilience, anxiety, depression, network analysis, gender differences, adolescents

## Abstract

**Objective:**

To explore the network structure of emotional resilience and anxiety-depressive symptoms in adolescents and identify gender differences in these networks.

**Method:**

A convenience sample of students from 21 middle schools in Jingzhou City was recruited for an online questionnaire survey. Emotional resilience, anxiety, and depression symptoms were assessed using the Adolescents’ Emotional Resilience Questionnaire, Generalized Anxiety Disorder Scale (GAD-7), and Patient Health Questionnaire (PHQ-9), respectively. Network analysis was performed to construct a model of emotional resilience dimensions (GP: generate positive emotion, RN: recover from negativity) and anxiety-depressive symptoms, with key features identified via expected influence (EI) and bridge expected influence (bEI). Gender differences were tested using the Network Comparison Test.

**Results:**

A total of 17,499 adolescents were included. The prevalence of anxiety (GAD-7 ≥ 10) was 7.43% in males and 13.32% in females; depression (PHQ-9 ≥ 10) prevalence was 9.89% in males and 16.33% in females. Core symptoms included “uncontrollable worry,” “depressed mood,” and “psychomotor problems,” while four bridge symptoms were identified: “GP,” “RN,” “restlessness,” and “depressed mood. “ Network structure (M=0.144, p < 0.001) and global strength (S=0.354, p < 0.001) differed significantly by gender.

**Conclusion:**

Adolescents need to develop emotional resilience in a balanced manner, and targeted interventions on core (e.g., “uncontrollable worry”, “psychomotor problems”) and bridge symptoms (e.g., “GP,” “RN,” “restlessness” and “depressed mood”) in the context of anxiety and depression are crucial for preventing these conditions in youth.

## 1 Introduction

The 2022 World Mental Health Report by the World Health Organization points out that over one billion people globally suffer from mental disorders, with depression and anxiety being the most common conditions (1). Depression and anxiety are common mental health disorders among adolescents. It is estimated that 6.2% of adolescents worldwide have depression disorders and 6.5% have anxiety disorders (2, 3). According to a meta-analysis of Chinese middle school adolescents, the detection rate of depressive symptoms was found to be 24.3% (4), and that of anxiety symptoms was 28.0% (5). Generalized anxiety disorder is characterized by persistent feelings of worry, restlessness, irritability, sleep disturbances, and nervousness, accompanied by symptoms such as palpitations, dry mouth, and sweating (6). Overall, anxiety disorders are the most common form of mental disorders among young people (7). A study on the prevalence and related disability burden of mental disorders among Chinese children and adolescents found that anxiety disorders represent the largest burden of mental disorders (8). Anxiety disorders not only significantly increase the risk of suicide among youth (9) but are also closely associated with the occurrence of substance use disorders and depression (10). Depression, a common and serious mental health disorder, is characterized by symptoms including low mood, anhedonia, lack of energy, and sleep disturbances (11). Global statistics show that 34% of adolescents are at risk of developing clinical depression (12). Depressive symptoms not only affect the mental health of adolescents but can also lead to declining academic performance, impaired cognitive function, and interpersonal relationship issues. Notably, studies have indicated a sharp rise in depression rates among young people, particularly within female populations (13, 14).

Most disorders among children and adolescents globally show significant gender differences (15). Significant changes in estrogen levels in adolescent girls may act as a trigger for the onset of depression (16). A recent study highlighted the unique challenges faced by adolescent girls, such as the influence of gender norms, academic pressure, and difficulties in peer relationships, making an important empirical contribution to understanding gender inequality in emotional distress and anxiety. It draws attention to the complex and interwoven nature of girls’ lived experiences (17). A meta-analysis by Salk et al. indicated that females generally report higher rates of depression diagnoses and symptoms than males, with gender differences peaking during adolescence, a trend observed universally across the globe (18). Similarly, studies within Chinese children and adolescents have found higher prevalence rates of depressive and anxiety disorders among girls compared to boys, highlighting significant gender differences (8, 19–21). Additionally, the comorbidity rate of depression and anxiety is higher in girls than in boys, with adolescents experiencing both conditions showing more severe depressive symptoms and behavioral issues (21). These findings underscore the critical role of adolescence in the emergence and progression of mental health problems, as well as the differential impact of gender on this process. A recent study on the network of psychological symptoms among Chinese high school students found that depression was the core symptom for boys, while anxiety was central for girls, indicating significant gender differences (22). However, recent meta-analyses reveal low treatment rates for mental disorders among children and adolescents, particularly for depression and anxiety disorders (23). Evidence suggests that targeted and directed prevention are more effective than universal prevention (14). Therefore, we hope that this study can provide some new insights into the prevention and early intervention of emotional problems in adolescents of different genders.

Resilience can be viewed as a defense mechanism, which enables people to thrive in the face of adversity, and improving resilience may be an important target for the treatment and prophylaxis of disease (24). The concept of resilience has evolved over time, yet there is considerable variation in how it is defined and measured across studies. Psychological resilience encompasses a broad range of dimensions; in the gradual refinement of this construct, some researchers argue that emotional resilience—although a significant component of psychological resilience—should be further distinguished from it (25, 26). The ability to generate positive emotions and recover quickly from negative emotional experiences is known as emotional resilience (26). This concept contains two key aspects, composed of two fundamental components: first, the ability to generate positive emotion when faced with negative emotional stimuli (GP); second, the ability to rapidly recover from negative emotional experiences even after they have been felt (RN). Some researchers suggest that GP and RN may relate to emotion regulation through distinct mechanisms (27). Previous studies have found that individuals with high emotional resilience recover more quickly from negative emotions and demonstrate better adaptive functioning and psychological well-being (28). Furthermore, Ong and colleagues found that positive emotional experiences help highly resilient individuals recover effectively from stress (29). When facing stressful events, positive emotions can enhance coping abilities, thereby improving psychological resilience and promoting mental and physical health (30). A longitudinal prospective sequential experimental cohort study on Canadian public safety personnel found that suicidal ideation and planning decreased directly after receiving Emotional Resilience Skills Training (31). However, it remains unknown whether GP or RN is more effective in reducing suicidal ideation.

By reviewing and synthesizing the literature on early research related to emotional resilience, it can be found that only a limited number of studies have provided conceptual definitions of emotional resilience. For example, a study involving 266 Chinese adolescents found that emotional resilience is negatively correlated with symptoms of depression and anxiety, and this negative correlation is partially mediated by positive emotions. The results highlight the role of emotional resilience in alleviating psychological problems and promoting mental health among Chinese adolescents (32). But very few studies have explored the relationship between emotional resilience and the dimensions of anxiety and depression symptoms, nor have they identified intervention targets (33). Previous studies have mostly relied on total scale scores to assess the relationship between resilience and anxiety or depression, overlooking the heterogeneity and complexity among the internal components of psychological variables, which makes it difficult to identify precise intervention targets. Furthermore, traditional statistical methods have limitations in handling the dynamic interactions among multidimensional variables, potentially obscuring the underlying associations between symptoms. In recent years, network analysis (NA) methods have become increasingly prevalent in the field of psychology. Network theory views mental disorders as the result of direct interactions between symptoms, with symptoms causally linked through biological, psychological, and social mechanisms. Symptoms act as nodes within psychopathological networks, with causal interactions between symptoms serving as connections between nodes (34). Numerous studies have utilized NA to explore the underlying mechanisms of mental disorders and identify symptoms that have more significant impacts (35–37). A previous study employed the NA method to explore the relationships among resilience, anxiety, and depression among clinical nurses at the item level (38). From a network perspective, symptoms are not interchangeable indicators. They are entities within a causal network, where their role depends on their position within that network. Theorists argue that highly “central” symptoms are more likely to propagate symptom activation throughout the network compared to those at the periphery (39). Therefore, these central symptoms are considered to play crucial roles in the onset and remission of mental disorders (39, 40).

Previous research has not explored, from a network perspective, the dimensions of adolescents’ emotional resilience and their relationships with anxiety and depression symptoms, nor is the influence of gender on the topological structure of the emotion resilience-symptom network well understood. To address this gap, this study innovatively applies network analysis to an integrated model of adolescent emotional resilience and anxiety-depression symptoms, aiming to answer the following key questions (1): How do the two dimensions of emotional resilience form a dynamically interconnected network with anxiety and depression symptoms? Which core nodes serve as “bridges” within this network? (2) Are there differences in network connectivity strength and core node distribution between male and female adolescents? Can these differences inform gender-specific interventions? By using network analysis to precisely delineate the mechanisms linking emotional resilience with anxiety and depression, this study aims to identify targeted intervention points for adolescent mental health problems, providing empirical support for developing personalized prevention and intervention strategies based on network characteristics.

## 2 Methods

### 2.1 Participants and study procedure

This is a study on the mental health status of adolescents, conducted by Jingzhou Mental Health Center in collaboration with the local Education Bureau from September 18 to October 8, 2023. The study adopted a convenience sampling method, distributing questionnaires in 21 middle and high schools in Jingzhou, China, with a methodology similar to previous studies (41). Data were collected through the “Questionnaire Star” platform embedded in WeChat. A QR code linked to the study introduction, invitation, and questionnaire entrance was designed and distributed. The research center provided the QR codes to cooperating schools and invited teachers at these schools to organize student and parent participation. After providing electronic written informed consent, participants used smartphones to scan the QR code and access the data collection form and questionnaire. A total of 19,686 questionnaires were distributed. Those who failed the attention-check questions, or whose completion times were excessively long or short, were excluded, resulting in 17,499 valid responses (an effective rate of 88.89%). This study has been approved by the Ethics Committee of Jingzhou Mental Health Center.

### 2.2 Measures

The Chinese version of the Patient Health Questionnaire-9 (PHQ-9) has demonstrated good reliability and validity in both general populations and youth samples (42, 43). This questionnaire was used to assess depressive symptoms over the past two weeks. The severity of anxiety symptoms was measured using the Chinese version of the 7-item Generalized Anxiety Disorder Scale (GAD-7) (44, 45), which has been validated among Chinese adolescents (46). Both scales use a 4-point Likert scoring system, where 0 indicates “not at all” and 3 indicates “nearly every day.” Following previous research (47, 48), a total score of 10 or higher on either the PHQ-9 or GAD-7 is considered “test-positive,” indicating possible presence of depression or anxiety. In this study, the Cronbach’s alpha coefficients for the PHQ-9 and GAD-7 were 0.91 and 0.93, respectively.

The Adolescents’ Emotional Resilience Questionnaire (AERQ) is an 11-item self-report measure of emotional resilience. See **Supplementary Table S1** for details. The questionnaire assesses two dimensions: the ability to generate positive emotion (GP) and the ability to recover from negative emotional experiences (RN). It is suitable for adolescents aged 11 to 20. A six-point Likert scale is used, ranging from 1 (“completely disagree”) to 6 (“completely agree”), with five items reverse-scored. The total emotional resilience score is obtained by summing the scores of the two dimensions. Higher scores indicate greater emotional resilience. The questionnaire has demonstrated good internal consistency and discriminant validity in previous studies, and is used to assess adolescents’ emotional resilience (27). In this study, the Cronbach’s alpha coefficient for the AERQ was 0.84; the internal consistency coefficients for the subscales were as follows: GP, 0.90; RN, 0.79.

### 2.3 Statistical analyses

#### 2.3.1 Network estimation

In psychometrics, accurate estimation of the number of dimensions has long been a challenging issue. Exploratory Graph Analysis (EGA), proposed by Golino and Epskamp (49), is an emerging dimension detection method in recent years. Based on the graphical lasso algorithm and using the EBIC criterion to determine the regularization parameter, EGA can assess the dimensional structure of items within psychological constructs. Overall, evidence suggests that compared to factor analysis—regarded as the “gold standard” for dimension assessment—EGA performs better in accurately detecting the correct number of dimensions in psychological scales, particularly when the sample size is large (50). In this study, we employed Exploratory Graph Analysis (EGA) to estimate the dimensionality of the GAD-7, PHQ-9, and AERQ, and evaluated model stability using the bootstrap method (bootEGA). In the final model, 27 nodes were distributed across four communities (**Supplementary Figure S1**), which was largely consistent with our pre-specified dimensional structure (one dimension for anxiety, one for depression, and two for emotional resilience, totaling four dimensions). The PHQ-9 and GAD-7 are designed to assess specific symptom clusters (corresponding to depressive and anxiety symptoms, respectively), with each item directly reflecting a unique, clinically identifiable symptom. Using individual items as nodes enables the capture of fine-grained associations between symptoms, aligning with the core objective of network analysis to explore interactions at the symptom level. Some scholars argue that resilience factors act as independent, additive network nodes that can weaken the connections between symptoms or the self-sustaining nature of symptoms, thereby preventing maladaptive shifts in the system (51). For the AERQ, our focus lies on the broader dimensions of emotional resilience represented by its subscales. The AERQ subscales are empirically derived constructs with demonstrated internal consistency. Therefore, using subscale total scores as nodes better reflects our interest in the question of “how resilience dimensions interact with specific anxiety and depression symptoms,” rather than focusing on individual resilience items.

Network analysis of the dimensions of emotional resilience and anxiety and depressive symptoms was conducted using the R software (Version 4.4.0), with the network model estimated via the EBICglasso method (36). Network visualization was performed using the “qgraph” package in R. Expected Influence (EI) was calculated to identify central (i.e., influential) symptoms within the network. Compared to traditional centrality indices (e.g., node strength), this approach is more suitable for networks containing both positive and negative edges (52). Nodes with higher Expected Influence in the network are considered more important. The bridge function from the R package networktools (Version 1.5.2) was used to compute bridge Expected Influence (bEI) in order to identify bridge symptoms. Compared to bridges with lower expected influence, those with higher bEI reflect a greater risk of spreading activation from one community to others (53). To identify bridge symptoms, an 80% threshold of the bridge Expected Influence value was used as the cutoff (54). In addition, the mgm package was used to estimate the predictability of each node. Predictability refers to the extent to which the variance of a given node can be predicted by the variances of its neighboring nodes in the network, and is represented visually by the area of the ring around each node in the network plot.

#### 2.3.2 Network stability

To assess the accuracy and stability of the observed network model, the R package bootnet (Version 1.6) (36) was used to perform 1000 bootstrap samples for each node. Using non-parametric bootstrapping, a new dataset with 95% confidence intervals (CIs) was generated to evaluate the accuracy of edge weights (40). Lower overlap of the confidence intervals indicates higher accuracy in edge weight estimation. The stability of centrality indices—Expected Influence and bridge Expected Influence—was assessed using the correlation stability (CS) coefficient. A CS coefficient greater than 0.25 suggests acceptable stability for node centrality, while a coefficient greater than 0.5 indicates good stability (36). Finally, non-parametric bootstrapping based on 95% confidence intervals was used to estimate the differences between each pair of edges or nodes. A confidence interval that includes zero indicates a statistically significant difference between the paired edges or nodes.

#### 2.3.3 Network comparison

Considering the moderating role of gender on anxiety and depressive symptoms in adolescents (55), the present study aimed to investigate whether there are differences in network characteristics between genders. The R package NetworkComparisonTest (Version 2.2.2) (56) was used to perform 1000 permutations to assess global network strength (the sum of absolute values of all edge weights) and network structure (the distribution of edge weights). Additionally, the Benjamini-Hochberg procedure was applied to evaluate the strength of each edge between the two networks while controlling for multiple comparisons.

## 3 Results

This study included a total of 17,499 adolescents, among whom 8,932 (51.04%) were male and 8,567 (48.96%) were female. There were 4,432 students from Grade 7 (25.33%), 3,317 from Grade 8 (18.96%), 3,176 from Grade 9 (18.15%), 2,118 from Grade 10 (12.10%), 2,546 from Grade 11 (14.55%), and 1,910 from Grade 12 (10.91%). The average age was 14.32 (± 1.63) years.

There was a gender difference in the positive rate for anxiety (GAD-7 ≥ 10): 7.43% for males and 13.32% for females. A similar trend was observed for depression (PHQ-9 ≥ 10), with positive rates of 9.89% for males and 16.33% for females. The scores for each item on the GAD-7 and PHQ-9 scales, as well as the two dimensions of the AERQ, are shown in **Table 1**.

**Table 1:** Descriptive statistics of measurement items.

Item	Item content	Mean (SD)	Expected influence^a^	Predictability
GAD1	Nervousness	0.61(0.79)	0.849	0.668
GAD2	Uncontrollable worry	0.44(0.74)	1.019	0.686
GAD3	Excessive worry	0.56(0.80)	0.905	0.681
GAD4	Trouble relaxing	0.51(0.80)	0.943	0.649
GAD5	Restlessness	0.33(0.65)	0.949	0.584
GAD6	Irritability	0.50(0.80)	0.931	0.678
GAD7	Fear of awful events	0.49(0.80)	0.710	0.535
PHQ1	Anhedonia	0.43(0.73)	0.833	0.526
PHQ2	Depressed Mood	0.53(0.78)	0.979	0.667
PHQ3	Sleep problems	0.48(0.82)	0.739	0.484
PHQ4	Fatigue	0.59(0.83)	0.907	0.586
PHQ5	Appetite problems	0.46(0.77)	0.730	0.425
PHQ6	Worthlessness	0.57(0.86)	0.768	0.575
PHQ7	Trouble concentration	0.40(0.74)	0.776	0.473
PHQ8	Psychomotor problems	0.28(0.63)	0.969	0.529
PHQ9	Suicide ideation	0.25(0.62)	0.612	0.461
GP	Ability to generate positive emotion	41.59(9.93)	-0.401	0.245
RN	Ability to recover from negative emotional experiences	19.86(6.16)	-0.354	0.241

The two dimensions of the Adolescent Emotional Resilience Questionnaire (AERQ): The ability to generate positive emotion (GP) and The ability to recover from negative emotional experiences (RN); PHQ-9, the 9-item Patient Health Questionnaire; GAD-7, 7-item Generalized Anxiety Disorder Scale; SD, standard deviation.

a The values of expected influence were raw data generated from the network.

### 3.1 Network structure

**Figure 1:**
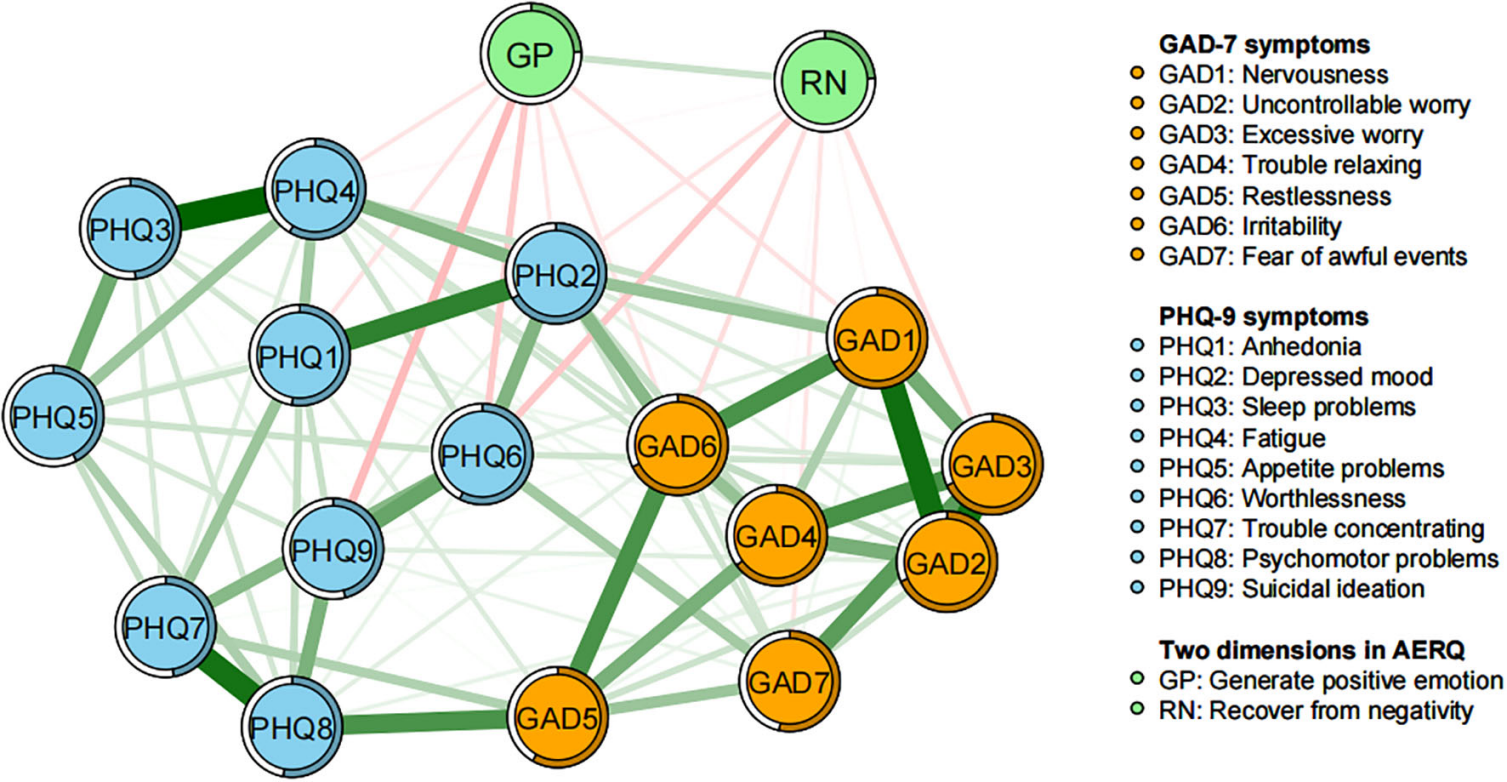
Network structure of emotional resilience and symptoms of anxiety and depression in adolescents. In the diagram, symptom nodes with stronger connections are closer to each other. The orange nodes denote the GAD-7 items; the sky-blue nodes denote the PHQ-9 items; the light green nodes denote the two dimensions of the AERQ. The dark green lines represent positive correlations, and the red lines represent negative correlations. To simplify the graph, edges with an absolute weight below 0.03 are not displayed. The edge thickness represents the strength of the association between symptom nodes.

The network structure of emotional resilience dimensions and anxiety and depressive symptoms for all participants is shown in **Figure 1**. Regarding the basic characteristics of the final network, first, 135 out of a possible 153 edges (88.24%) were non-zero, reflecting considerable interconnectivity between symptoms. The predictability of each symptom is depicted by ring-shaped pie charts (**Figure 1**, **Table 1**). The average node predictability was 0.539, indicating that 53.9% of the variance of nodes within the network can be explained by their neighboring nodes.

**Figure 2:**
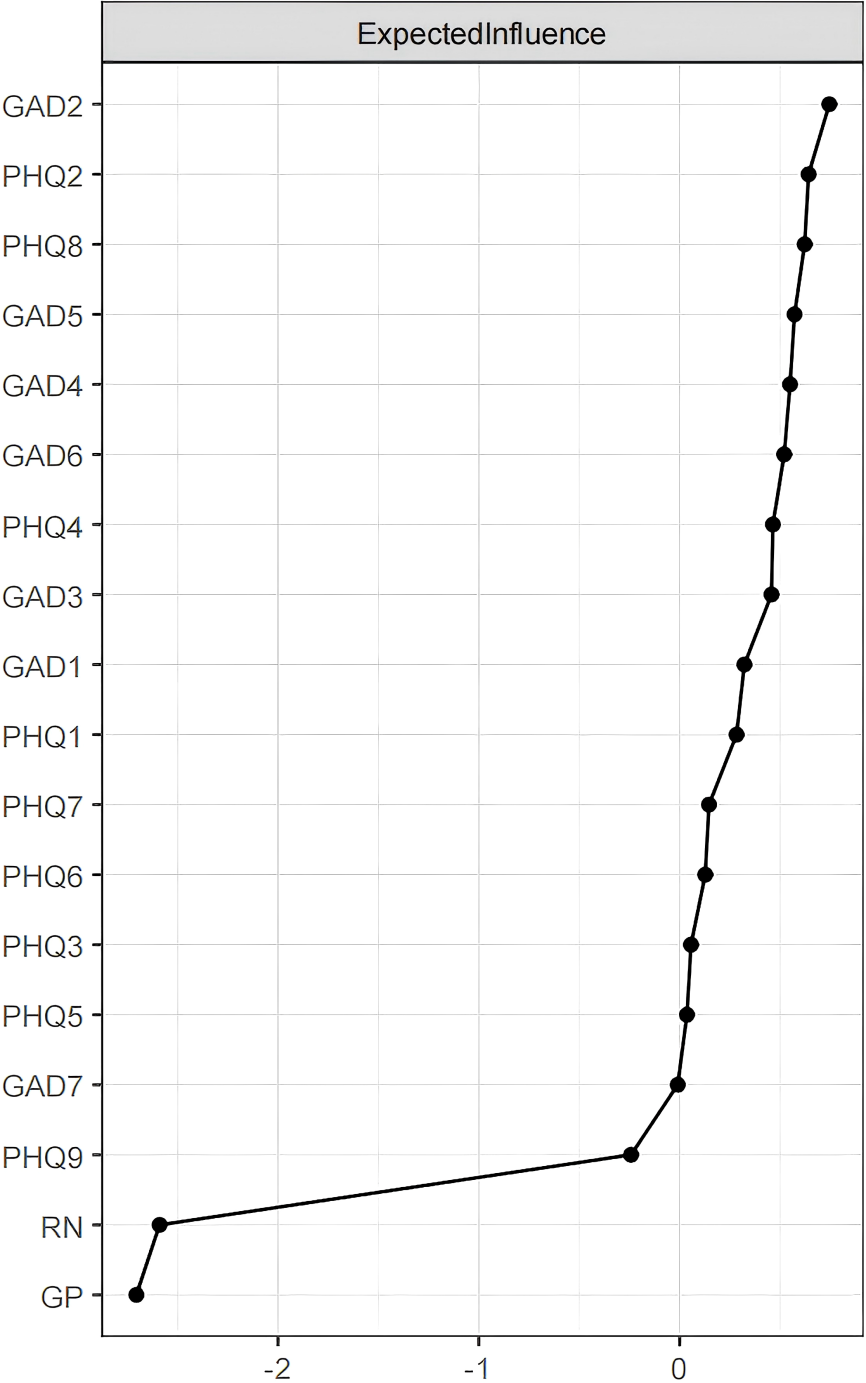
Centrality indices of the network structure for emotional resilience and symptoms of anxiety and depression in adolescents: Expected Influence. Centrality indices are shown as standardized z-scores.

“Uncontrollable worry” (GAD2), “depressed mood” (PHQ2), “psychomotor problems” (PHQ8), and “restlessness” (GAD5) occupy central positions in the node Expected Influence metrics, showing significant connectivity and predictive power (**Figure 2**, **Table 1**). This indicates that these individual symptoms are crucial and have the most substantial impact for understanding the structure of the emotional resilience and anxiety-depression network model. They may represent core symptoms requiring intervention, suggesting that improving these core symptoms could be more effective in alleviating the entire symptom network. This approach aligns with clinical strategies that prioritize addressing key symptoms.

As shown in **Figure 1**, the two dimensions of emotional resilience are negatively correlated with most anxiety and depression symptoms. The top three symptoms that have the strongest negative correlations with the ability to generate positive emotion (GP) are: “suicidal ideation” (PHQ9), “worthlessness” (PHQ6), and “nervousness” (GAD1) (with weights of -0.082, -0.069, and -0.049, respectively). For the ability to recover from negative emotional experiences (RN), the top three symptoms with the strongest negative correlations are: “worthlessness” (PHQ6), “excessive worry” (GAD3), and “irritability” (GAD6) (with weights of -0.071, -0.056, and -0.052, respectively).

**Figure 3:**
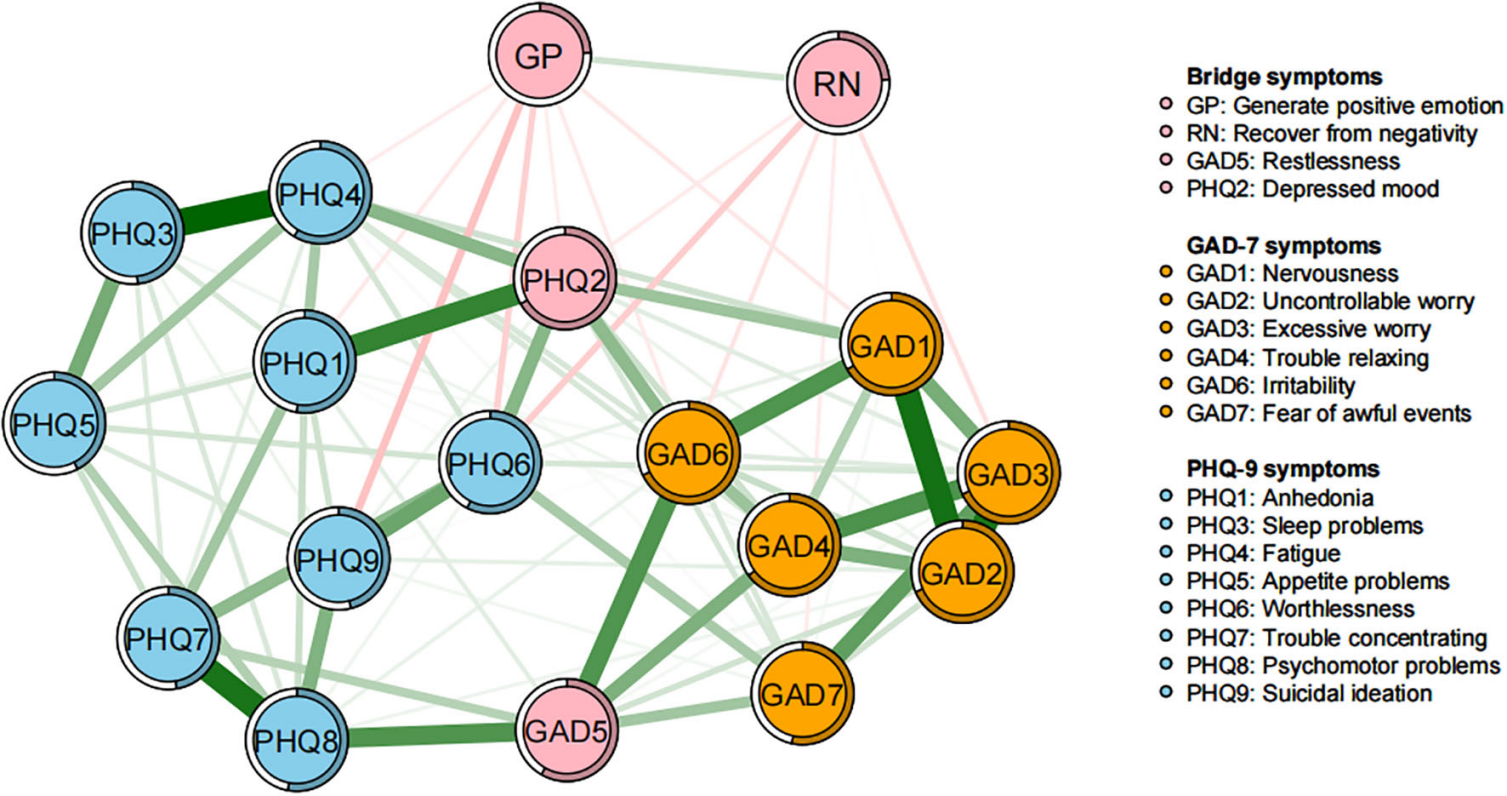
Bridge network structure of emotional resilience and symptoms of anxiety and depression in adolescents. In the diagram, symptom nodes with stronger connections are closer to each other. The orange nodes denote the GAD-7 items; the sky-blue nodes denote the PHQ-9 items; the light pink nodes denote the Bridge symptoms. To simplify the graph, edges with an absolute weight below 0.03 are not displayed.

**Figure 4:**
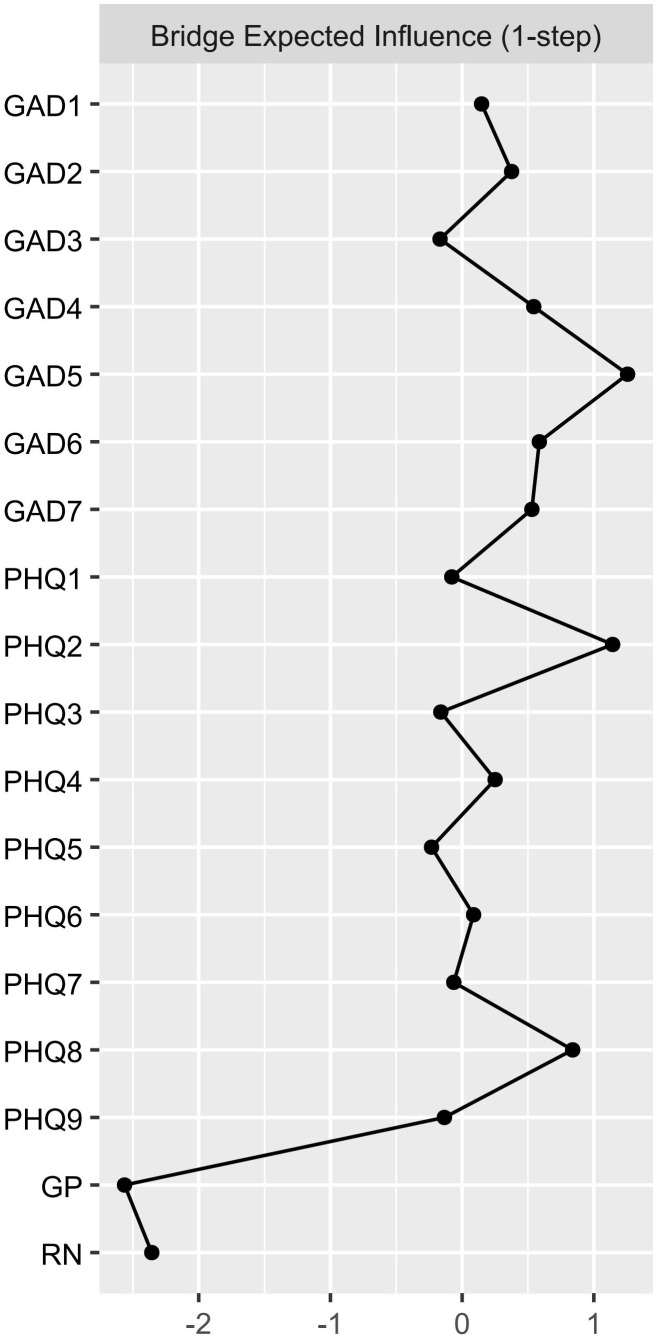
Bridge expected influence (1-step) of the Network Structure of Adolescent Emotional Resilience, Anxiety, and Depression Symptoms. Centrality indices are shown as standardized z-scores.

The study identified four bridge symptoms: “generate positive emotion,” “recover from negativity,” “restlessness,” and “depressed mood” (**Figures 3**, **4**). These bridge symptoms play a critical role in connecting different parts of the network, indicating their significance in the interaction between emotional resilience and anxiety and depression symptoms.

### 3.2 Network stability and accuracy

**Figure 5:**
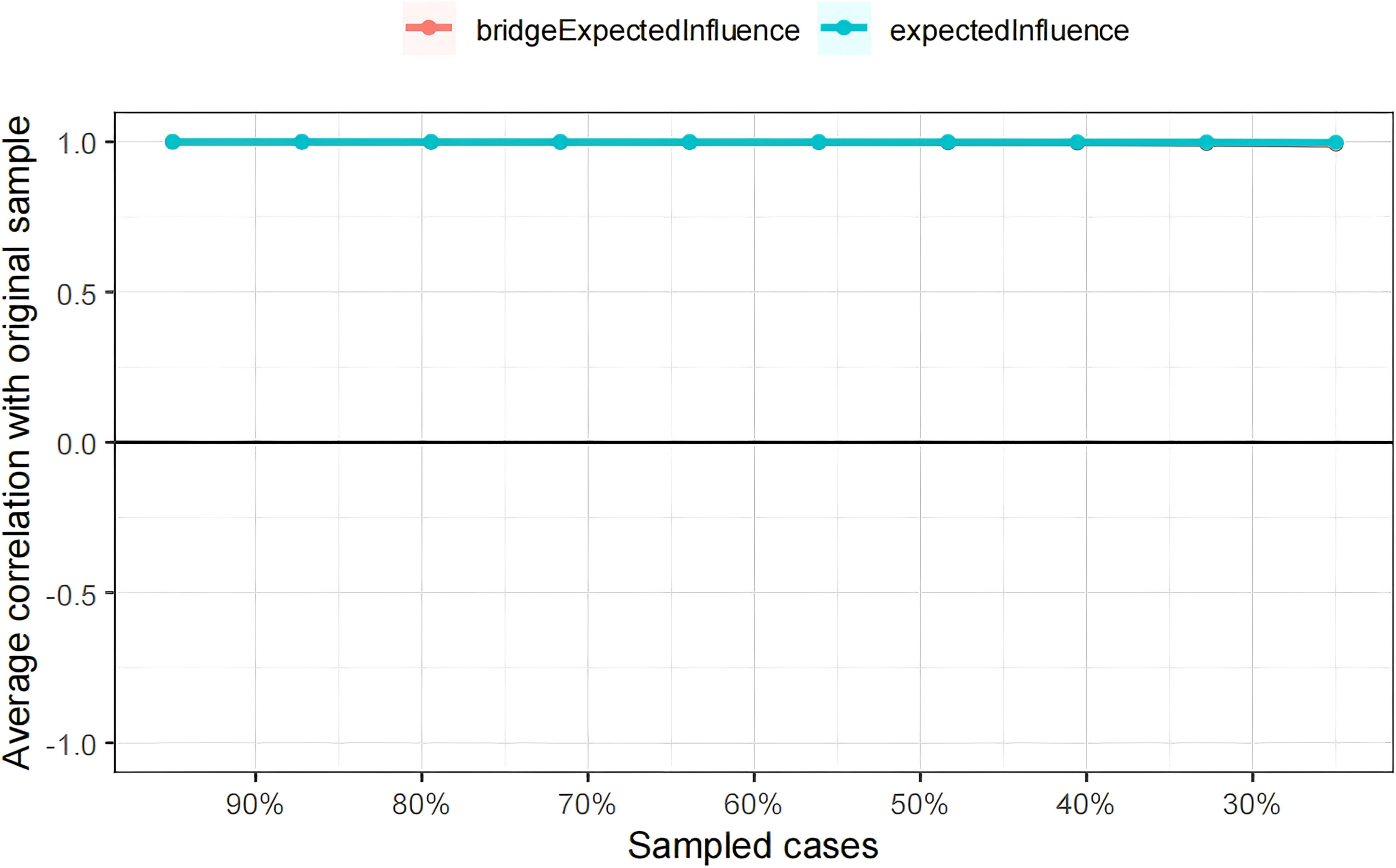
Stability of centrality and bridge centrality indices using case-dropping bootstrap. The x-axis represents the percentage of cases of the original sample used at each step. The y-axis represents the average of correlations between the centrality indices in the original network and the centrality indices from the re-estimated networks after excluding increasing percentages of cases. The line indicates the correlations of EI and bEI.

Both the Expected Influence and bridge Expected Influence values demonstrated excellent stability (with CS coefficients of 0.75), indicating that the network structure would not substantially change even when 75% of the sample was removed (**Figure 5**). Additionally, the 95% confidence intervals (CIs) obtained through bootstrapping were narrow, suggesting reliable estimates for the edge weights (**Supplementary Figure S2**). Non-parametric bootstrap results also showed that most comparisons of node EI and bEI were statistically significant (**Supplementary Figures S3**, **S4**).

### 3.3 Network comparison tests

**Figure 6:**
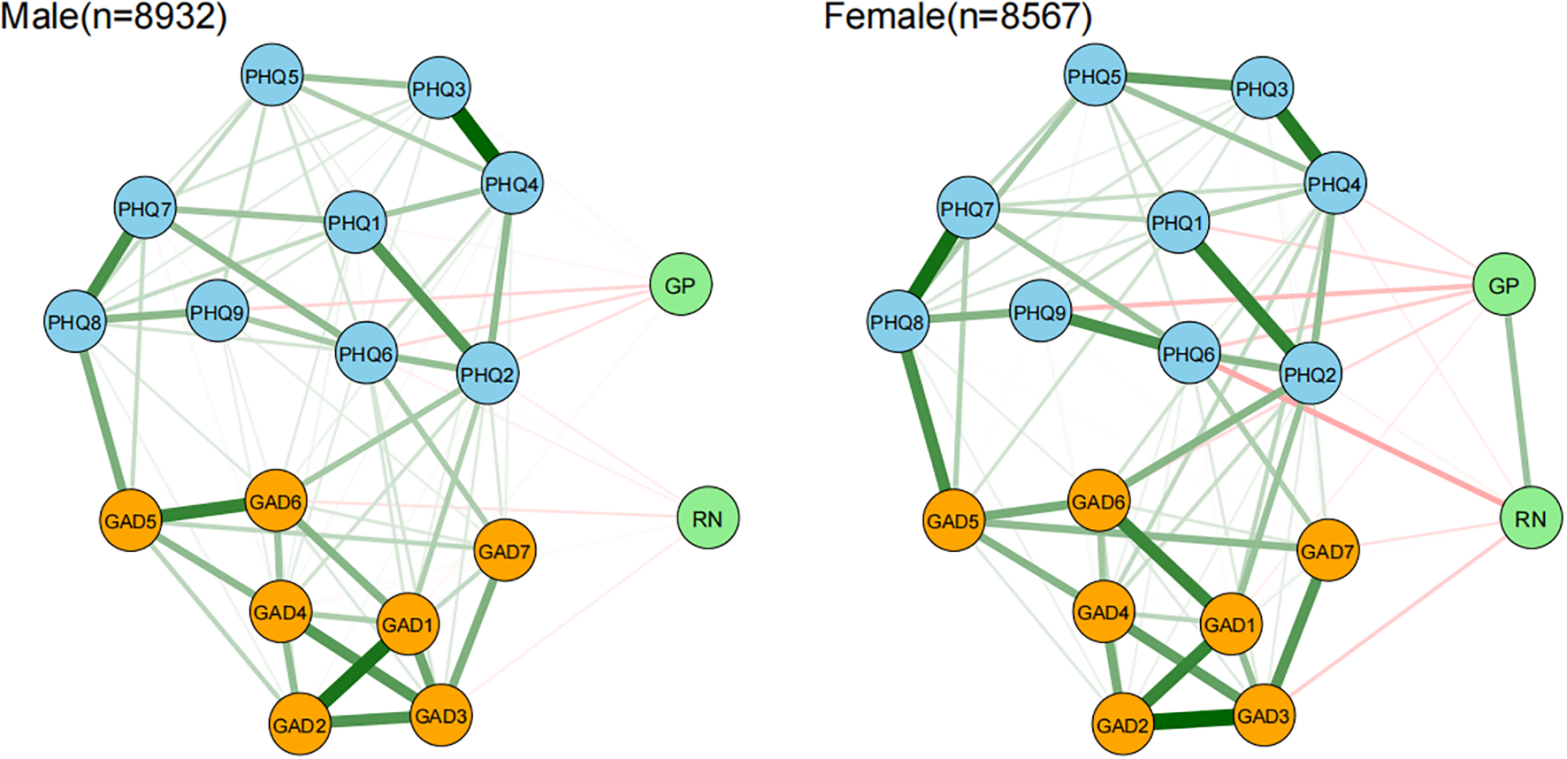
Network comparison of emotional resilience and symptoms of anxiety and depression between adolescent males and females. In the diagram, symptom nodes with stronger connections are closer to each other. The orange nodes denote the GAD-7 items; the sky-blue nodes denote the PHQ-9 items; the light green nodes denote the two dimensions of the AERQ. To simplify the graph, edges with an absolute weight below 0.03 are not displayed.

Network comparisons of gender differences in emotional resilience with anxiety and depressive symptoms are presented in **Figure 6**. Further analyses revealed significant gender differences in both network structure invariance (M=0.144, p < 0.001) and global strength (females: 8.415, males: 8.061; S=0.354, p < 0.001) (**Supplementary Figure S5**), suggesting that emotional resilience has a more pronounced inhibitory effect on anxiety and depression in females. Results of edge invariance tests indicated that there were multiple edges with significantly stronger strength in the female group (p < 0.05), including those within the anxiety-depression dimensions: “Uncontrollable worry” (GAD2) and “excessive worry” (GAD3), “worthlessness” (PHQ6) and “suicidal ideation” (PHQ9); that within the emotional resilience dimension: GP-RN; and that across dimensions: RN and “worthlessness” (PHQ6). This result reflects greater connectivity and stronger inhibitory effects of emotional resilience in these dimensions among females. Centrality invariance tests showed gender differences in bridge Expected Influence for nodes RN and “restlessness” (GAD5) (p < 0.05). Specifically, GAD5 exhibited a stronger bridging role in the female network, while RN had a more significant inhibitory effect on anxiety and depression in females. In addition, most other nodes did not show significant differences between the gender-based networks (p ≥ 0.05).

## 4 Discussion

This study is the first to apply network analysis methods to explore the dynamic associations between emotional resilience and anxiety and depressive symptoms among adolescents. “Uncontrollable worry” emerged as the most central symptom in the entire network, followed by “depressed mood,” “psychomotor problems,” and “restlessness.” Therefore, the activation or persistence of these symptoms may trigger or sustain the dynamic development of other anxiety and depressive symptoms. Additionally, in this sample, the bridge symptoms connecting emotional resilience with anxiety and depressive symptoms can be categorized into two types: protective factors (“recover from negativity,” “generate positive emotion”) and risk factors (“restlessness,” “depressed mood”). This study reveals the complex dynamic interplay between emotional resilience and symptoms of anxiety and depression among adolescents, highlights the role of gender differences, and offers a novel perspective and empirical support for understanding the network mechanisms underlying adolescent mental health.

Notably, “uncontrollable worry” and “depressed mood” exhibited the highest Expected Influence values in the network, which is consistent with findings from a study by Su et al. based on a large sample of early adolescents in China (N=15,391) (57). This consistency suggests that these two emotional symptoms may serve as key hubs within the anxiety-depression network across different adolescent age groups, playing a central role in symptom transmission and persistence.

“Uncontrollable worry” exhibits the strongest connectivity and predictive power in the network. The metacognitive model of generalized anxiety disorder places worrying, meta-worry (“worry about worry”) and corresponding underlying metacognitive beliefs (i.e., beliefs about worry) as central in the maintenance of symptoms (58). Psychological resilience has an inhibitory effect on worry-related factors (59), providing a target for clinical interventions.

In this study, “depressed mood” was observed as a core and bridge symptom within the network among non-clinical adolescents. This finding resonates with the centrality characteristics of depressive mood observed by Beard et al. (60) in clinical samples, suggesting a developmental stage correspondence—both studies indicate that depressive mood exhibits significant centrality characteristics regardless of whether the sample consists of clinical or non-clinical populations. This aligns with existing research indicating that depressive mood serves as a central pathological mechanism in depression (61).

Another bridge symptom identified in this study is “restlessness.” Interestingly, in our sample, both “psychomotor problems” and “restlessness” were also core symptoms within the network. Additionally, the bridging effect of “restlessness” was stronger in females, consistent with previous studies (37, 62), suggesting that physiological anxiety symptoms (such as psychomotor agitation) may be a critical pathway for the transdiagnostic transmission of emotional disorders in females. The findings of this study align with previous research indicating gender differences in somatic experiences; for example, Ruchkin and Schwab-Stone noted in a longitudinal study of urban adolescents that females exhibit higher levels of somatic symptoms and somatic anxiety compared to males (63). These gender differences may stem from interactions between biological susceptibility, psychological coping mechanisms, and sociocultural pressures. From a clinical perspective, given their transdiagnostic nature, targeted interventions for bridge symptoms are likely to be effective for both co-occurring disorders. This suggests that interventions focused on regulating psychomotor activity—such as mindfulness-based therapies, relaxation exercises, or behavioral activation therapy—would be particularly effective, especially for patients with comorbid conditions. Recent research indicates that behavioral activation therapy has shown stronger effects compared to Acceptance and Commitment Therapy (64).

The two dimensions of emotional resilience (GP, RN) served as bridge nodes in the network and demonstrated broad negative associations with both anxiety and depressive symptoms. These findings align with prior research (38, 65–67), which has established resilience as a protective factor against the development of anxiety and depression. Notably, GP exerted the strongest inhibitory effect on “suicidal ideation,” whereas RN showed the most robust negative association with “worthlessness.” Moreover, the negative association between RN and “worthlessness” was stronger in females, suggesting that females’ emotional recovery ability may be more susceptible to the influence of self-evaluation. When emotional recovery capacity is insufficient, females are more likely to fall into depression following setbacks in self-worth. Cognitive reappraisal can mitigate the impact of depressive symptoms on emotional reactivity in adolescents, particularly showing significant effects in emotional recovery (68). Resilient individuals also utilize positive emotions to recover from negative emotional experiences (69). Higher emotional resilience enables individuals to experience greater positive emotions, which fosters more oppositional and negative attitudes toward suicidal ideation. Positive emotions not only buffer the negative impacts of crises but also reduce depressive feelings and suicidal thoughts, promoting psychological growth (70), aligning with the broaden-and-build theory of positive emotions. This theory posits that over time, the broadening effects initiated by positive emotions build enduring personal resources (71, 72). Emotional recovery ability refers to the capacity to rapidly bounce back from negative emotional experiences; by accelerating the resolution of negative emotions, it helps alleviate core depressive symptoms such as low self-evaluation.

Gender difference analyses revealed that the female symptom network exhibited higher global strength and several gender-specific connections. Notably, females showed stronger connectivity between GP and RN, suggesting a greater reliance on the synergistic effects of emotional resilience to resist symptom development. In the female network, stronger connections were observed for GAD2–GAD3 and PHQ6–PHQ9, which may be linked to a higher tendency among females to engage in rumination—internally processing emotions through repetitive thinking or self-critical behaviors (73, 74). This aligns with Nolen-Hoeksema’s theory of gender differences in rumination, which posits that females are more prone to repetitive worry, partially explaining why they are at higher risk for depression and anxiety (75, 76). Our findings contrast with those of Cai et al. (77), who investigated the network structure of depressive and anxiety symptoms in adolescents (n = 1057) during the later phase of the COVID-19 pandemic and found no significant gender effect on network structure. This discrepancy may stem from differences in sample timing, population characteristics, or modeling approaches.

Recent meta-analytic results indicate that psychotherapy leads to significant improvements in overall functioning among adolescents with anxiety (78), and is also effective in preventing subclinical symptoms from developing into more severe depression. Psychological interventions have shown broad applicability across different populations (79). This study provides important implications for adolescent mental health interventions: for adolescents primarily presenting core symptoms such as “uncontrollable worry” and “depressed mood,” targeted intervention strategies can be designed. For example, cognitive behavioral therapy (CBT) techniques such as “worry behavior prevention” can be combined with mindfulness training to reduce hyperarousal. Given females’ higher tendency toward rumination and greater emotional connectivity, interventions targeting worry, meta-worry, and underlying metacognitive beliefs—such as employing “metacognitive therapy” to interrupt excessive focus on worry—may have a significant impact on both anxiety and depressive symptoms in individuals with generalized anxiety disorder (58).

In addition, psychological interventions should prioritize enhancing GP and RN in adolescents, particularly female adolescents, to strengthen their resilience to depression and anxiety and break the vicious cycle of negative cognition and emotion. One study found that higher resilience can act as a protective factor against mood disorders (80). Notably, interventions such as resilience training and CBT have been shown to effectively enhance psychological resilience (81). Mindfulness training has been proven to improve resilience (82), with stronger effects observed in females than in males (83). Another study indicated that emotional resilience in females demonstrates a more pronounced inhibitory effect on negative emotional symptoms. According to the mindfulness-based stress reduction model, after engaging in positive cognitive evaluation, individuals experience an expanded attentional scope, reframe their understanding of stressful events, and ultimately increase their resilience levels (84). It is recommended that mindfulness strategies be tailored based on gender differences: females may benefit more from emotion awareness training, while males may respond better to active interventions such as mindful yoga or tai chi, which align with their externalizing coping styles (85). Furthermore, a recent systematic review found that interventions combining CBT with mindfulness techniques appear to have a positive impact on individual resilience (86). Researchers have also suggested that educators and policymakers should encourage adolescents to engage in regular physical activity. Exercise not only directly reduces emotional distress such as anxiety and improves overall well-being but also indirectly promotes mental health by fostering resilience (87, 88). Physical activity is an effective factor in enhancing stress resistance, with particularly significant effects observed in females (89).

Both internal assets and external resources may serve as key factors in enhancing resilience (90). Strengthening collaboration among schools, families, and communities can better support adolescent mental health. A study involving 163 children aged 8 to 10 found that mindfulness instruction in schools or other settings helps improve children’s emotional resilience (91), and therefore the application of this teaching approach should be encouraged. Research indicates that school-based universal interventions focusing on resilience enhancement are promising and can at least short-term alleviate symptoms of depression and anxiety in adolescents, especially when using cognitive behavioral therapy (CBT)-based approaches (92). A study by Rudolph et al. (93) showed that adolescent females who participated in a single-session emotional mindset course (E-MIND) experienced improved emotional states, increased self-efficacy in emotion regulation, and enhanced use of coping strategies. Single-session interventions are low-cost, time-efficient, and easy to implement, making them suitable for resource-limited settings. If integrated into school health education and combined with parental and teacher involvement, the effectiveness may be further enhanced. Internationally validated school-based mental health programs such as My FRIENDS Youth have been shown to significantly improve adolescent emotional resilience (94, 95). Through cognitive behavioral training, this program effectively alleviates emotional symptoms in adolescents, particularly showing notable benefits for anxiety reduction in females, offering an adaptable model for reducing the burden of mental disorders in other countries. Additionally, a systematic review and meta-analysis by Schäfer et al. (96) found that online and mobile interventions are as effective as face-to-face interventions. Digital resilience interventions hold potential for addressing future mental health challenges; they can promote psychological well-being, enhance resilience components, and possibly help maintain good psychological functioning or enable rapid recovery following periods of stress. Based on these findings, it is recommended to develop digital interventions aimed at enhancing psychological resilience.

However, several limitations should be noted. First, due to the cross-sectional design, the causal direction among symptoms cannot be inferred. Future studies could further examine the causal relationships between variables using cross-lagged panel models or intervention designs. Second, the sample was drawn from a single region and recruited via convenience sampling, which may introduce regional, cultural, and selection biases. Specifically, although convenience sampling provided practical advantages for this study—such as ease of data collection and resource efficiency—it may have led to an overrepresentation of easily accessible groups, such as adolescents from specific schools, thereby limiting the generalizability of the findings to the broader target population. This could reduce the external validity of the results across diverse cultural contexts, geographical populations, and non-clinical adolescents. Therefore, future research should replicate and validate these findings in diverse cultural and regional settings, with particular attention to gender differences in resilience and emotional symptoms within collectivistic cultures. Additionally, probability sampling methods are recommended to enhance sample representativeness and the external validity of the findings. Third, data were collected using self-report questionnaires, which may be subject to potential biases such as recall bias and response bias. These limitations suggest that future research combining longitudinal designs, cross-cultural comparisons, and multimodal assessment approaches (e.g., incorporating objective physiological indicators or observer ratings) would help overcome the current constraints and provide more universally applicable theoretical foundations for the precise intervention of adolescent emotional health.

In summary, this study based on network analysis reveals the dynamic associations between emotional resilience and anxiety and depressive symptoms in adolescents, as well as the gender differences underlying these relationships. Core symptoms such as “uncontrollable worry” and “psychomotor problems,” along with bridge symptoms including “GP,” “RN,” “restlessness,” and “depressed mood,” represent potential intervention targets. The findings indicate that gender differences should be fully considered in adolescent mental health interventions, necessitating more targeted strategies. For females, interventions should focus on strengthening RN and blocking the cross-system transmission of somatic anxiety symptoms, while for males, cognitive behavioral therapy and active mindfulness training may be particularly effective. Moreover, multi-institutional collaboration is needed, with governments, schools, and healthcare institutions working together to explore effective measures to enhance adolescents’ emotional resilience. This would contribute to more effective prevention and intervention of emotional problems and promote psychological health during adolescence.

## Data Availability

The raw data supporting the conclusions of this article will be made available by the authors, without undue reservation.

## Supplementary material

Supplementary Figure 1 Dimensionality results from EGA (left) and item stability of the GAD-7, PHQ-9, and AERQ (right).

Supplementary Figure 2 Bootstrapped confidence intervals of edge weights.

Supplementary Figure 3 Bootstrapped stability test for ‘expected influence’.

Supplementary Figure 4 Bootstrapped stability test for’bridge expected influence’.

Supplementary Figure 5 Difference in global strength and maximum of difference between adolescent males and females. There was a significant difference in network global strength between females and males (females: 8.415, males: 8.061; S=0.354, p < 0.001), as well as in network structural invariance (M=0.144, p < 0.001).
