# Supplementary figures and images for: Network characteristics of emotional resilience, anxiety, and depression among Chinese adolescents and their gender differences
Source: Front Psychiatry. 2025 Sep 16;16:1651506. doi: 10.3389/fpsyt.2025.1651506 (PMC12479411; doi:10.3389/fpsyt.2025.1651506)

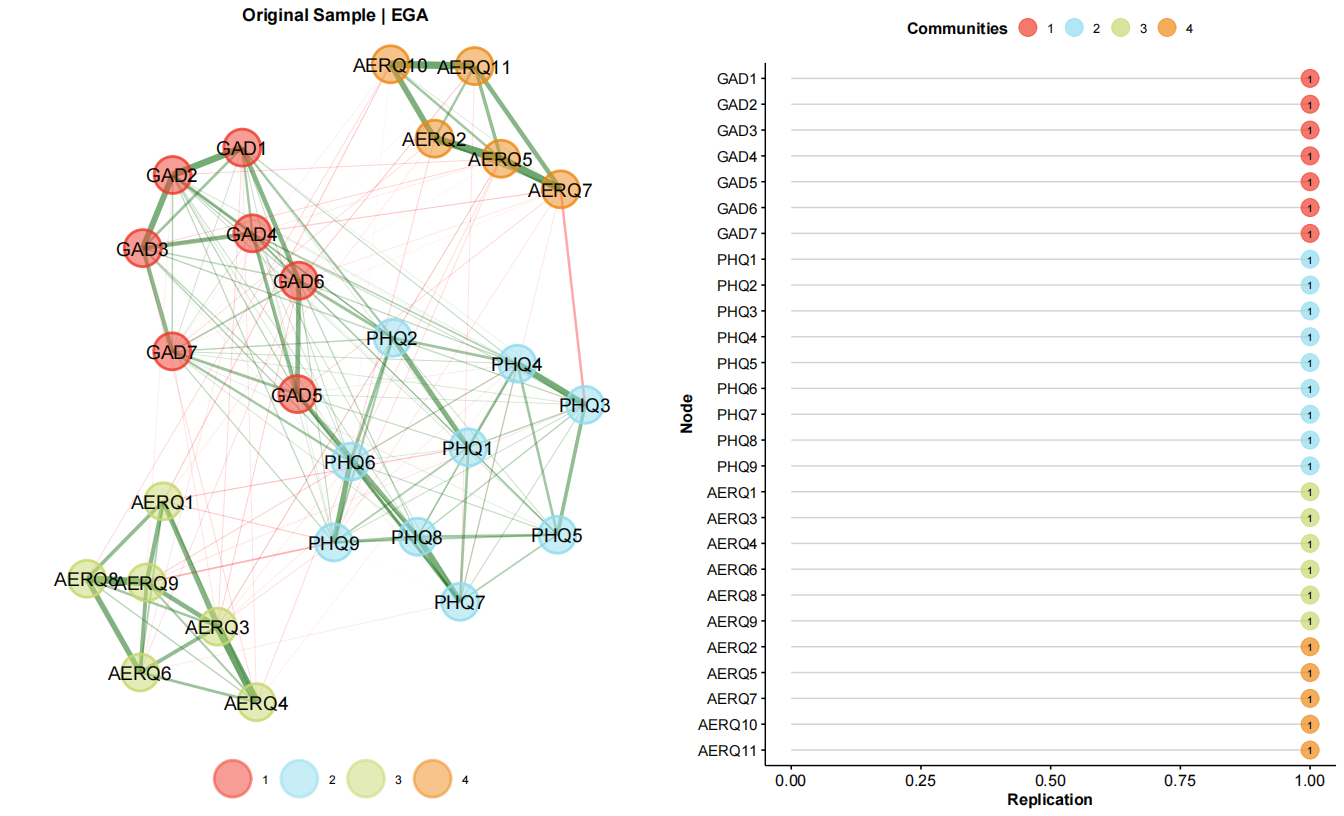

Supplement: Supplementary Figure 1 — Dimensionality results from EGA (left) and item stability of the GAD-7, PHQ-9, and AERQ (right). [file Image1.tif]

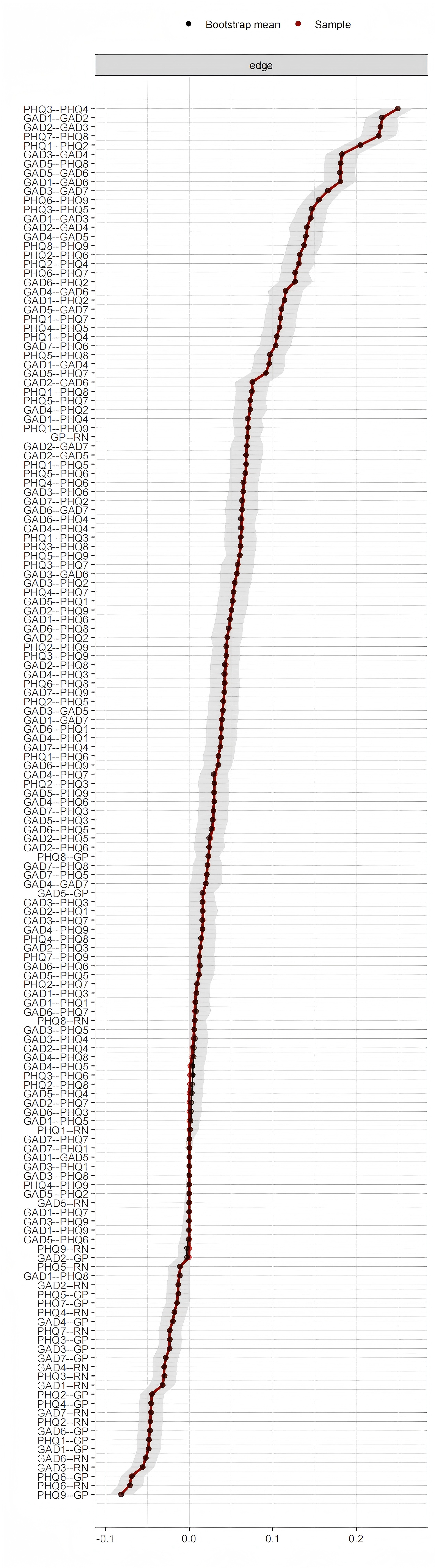

Supplement: Supplementary Figure 2 — Bootstrapped confidence intervals of edge weights. [file Image2.jpeg]

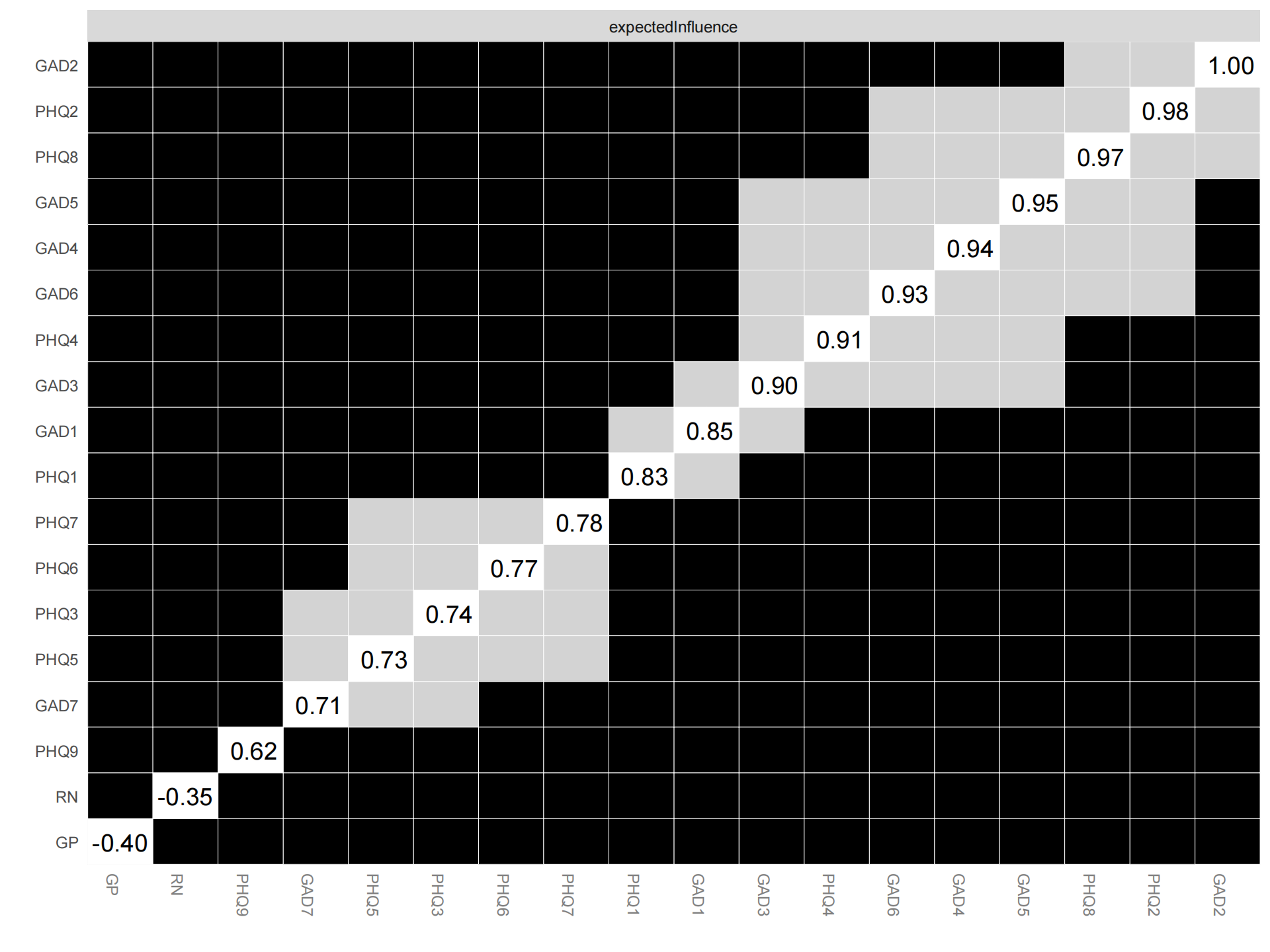

Supplement: Supplementary Figure 3 — Bootstrapped stability test for ‘expected influence’. [file Image3.tif]

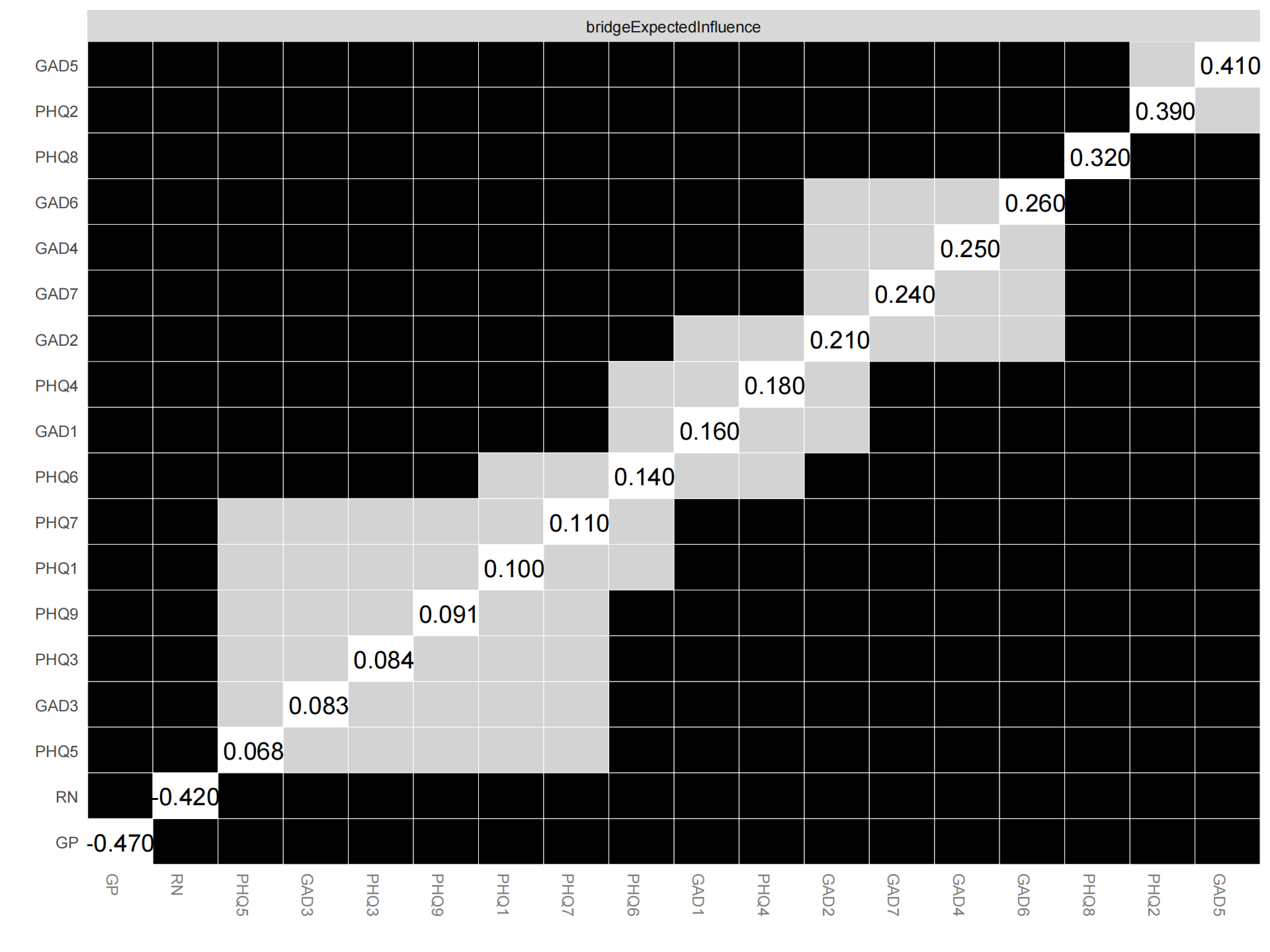

Supplement: Supplementary Figure 4 — Bootstrapped stability test for’bridge expected influence’. [file Image4.tif]

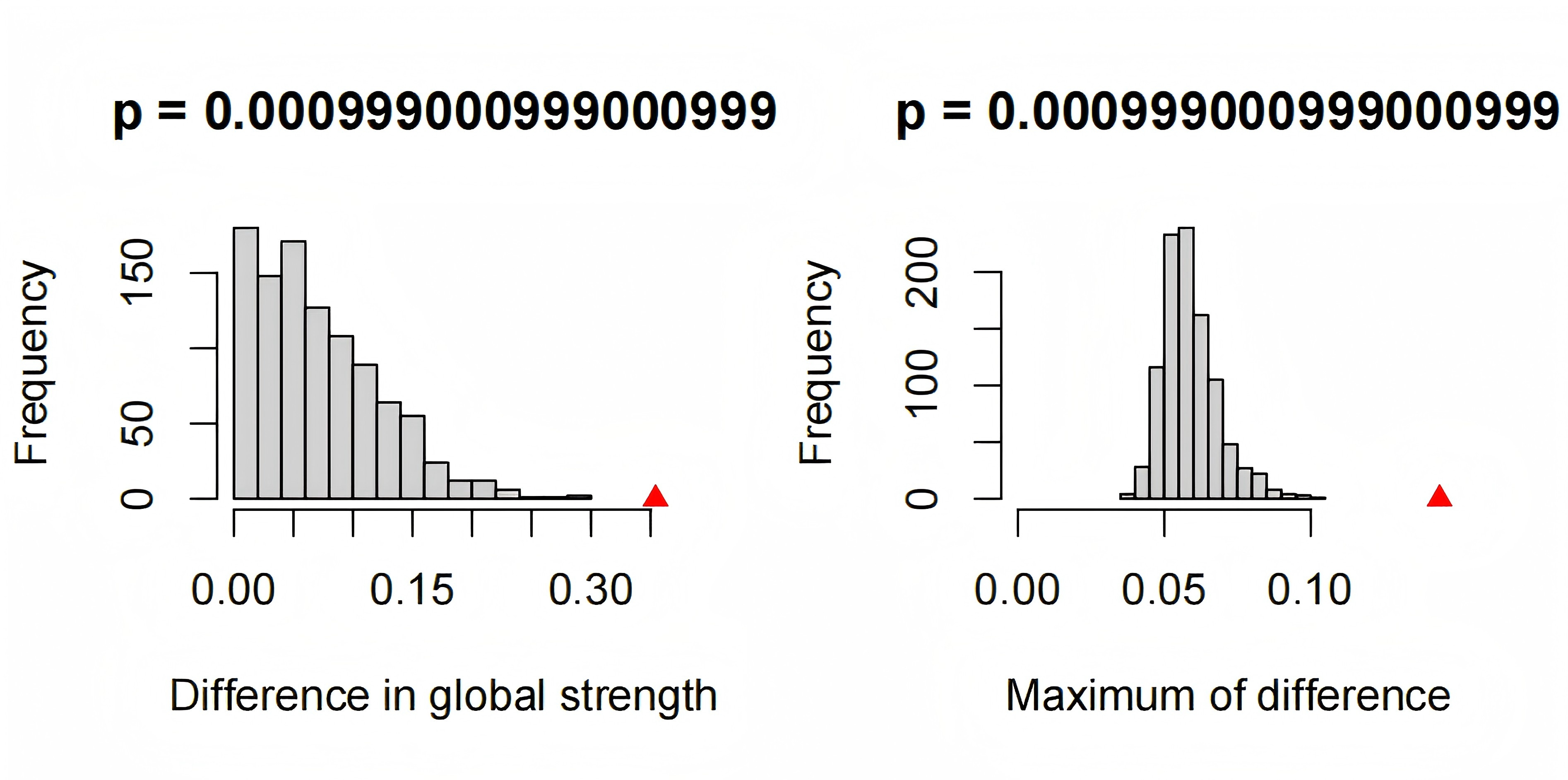

Supplement: Supplementary Figure 5 — Difference in global strength and maximum of difference between adolescent males and females. There was a significant difference in network global strength between females and males (females: 8.415, males: 8.061; S=0.354, p < 0.001), as well as in network structural invariance (M=0.144, p < 0.001). [file Image5.jpeg]
