# Supplementary material for: Network characteristics of emotional resilience, anxiety, and depression among Chinese adolescents and their gender differences
Source: Front Psychiatry. 2025 Sep 16;16:1651506. doi: 10.3389/fpsyt.2025.1651506 (PMC12479411; doi:10.3389/fpsyt.2025.1651506)
Supplement: Supplementary file 6 [file Table1.docx]

Table S1. Adolescents’ emotional resilience questionnaire (in Chinese)

| ****Item number**** | ****Item content**** |
| --- | --- |
| 1 | When I’m in a bad mood, I can think of something happy. |
| 2 | Criticism from others will make me sad for a long time. |
| 3 | No matter what difficulties I meet, I can keep a good mood. |
| 4 | My mood is not easily disturbed by the outside world. |
| 5 | Unhappy things will make me upset for a long time. |
| 6 | I can adjust my negative emotions in a short time. |
| 7 | Unpleasant events during the day often keep me awake at night. |
| 8 | In the face of stress or frustration, I can find my own comfort. |
| 9 | No matter how bad I feel, I can always look on the bright side. |
| 10 | I feel bad when people misunderstand me. |
| 11 | It is hard for me to calm down after an argument. |

The ability to generate positive emotions: 1, 3, 4, 8, 9. The ability to recover from negative emotional experiences: 2*, 5*, 6, 7*, 10*, 11* (* reverse scoring).
